# Multivalvular destruction as the primary presentation of aggressive infective endocarditis with subaortic valve membrane

**DOI:** 10.12669/pjms.37.2.2798

**Published:** 2021

**Authors:** Sultan Abdulwadoud Alshoabi, Nouradden Noman Aljaber, Moawia Bushra Gameraddin, Awatef Mohammed Omer

**Affiliations:** 1 Sultan Abdulwadoud Alshoabi, Department of Diagnostic Radiology Technology, College of Applied Medical Sciences, Taibah University, Almadinah Almunawwarah, Kingdom of Saudi Arabia; 2 Nouradden Noman Aljaber, Department of Medicine, Faculty of Medicine, Sana’a University, Sana’a, Republic of Yemen; 3 Moawia Bushra Gameraddin, Department of Diagnostic Radiology Technology, College of Applied Medical Sciences, Taibah University, Almadinah Almunawwarah, Kingdom of Saudi Arabia; 4 Awatef Mohammed Omer, Department of Diagnostic Radiology Technology, College of Applied Medical Sciences, Taibah University, Almadinah Almunawwarah, Kingdom of Saudi Arabia

**Keywords:** Aortic regurgitation, Mitral regurgitation, Infective endocarditis, TTE, TEE

## Abstract

Multivalvular destruction may be a clinical manifestation of infective endocarditis (IE), which is a devastating infection of the heart either alone or superimposed with congenital subaortic membrane as in this case report. Here, we report a case of multivavular destruction with severe vegetation presented as a manifestation of infective endocarditis (IE) in a neglected case of 18-year-old male with previous rheumatic heart disease. Transesophageal echocardiography is an important imaging modality for diagnosis of superimposed aortic and heart lesions. Early necessary investigation and correct diagnosis is mandatory to prevent bad complications.

## INTRODUCTION

Infective endocarditis (IE) is an infection of a heart valve that can affect a native or prosthetic valve. It is a devastating disease with a 30% mortality rate at one year.^1^ More than 50% of cases occur in persons with no known heart disease. IE is a feared disease in cardiology with a heterogeneous etiology, symptoms, signs, and course. Although, the causative organisms vary, staphylococcus aureus bacteria are reported as the most common causative organism.^1^,^2^ Risk factors of IE include patients with congenital heart disease, patients on hemodialysis, and immunocompromised patients.^3^ Predisposing factors for IE include prior IE, mitral valve prolapse, and bicuspid aortic valve.^4^ Echocardiography including both 2D trans-thoracic echocardiography (TTE) and trans-esophageal echocardiography (TEE) have complementary role in diagnostic imaging in IE.^5^ The diagnostic value of non-invasive imaging modalities is still unclear.^6^ In this study, we report a strange case of IE that was primarily diagnosed as rheumatic heart disease at the onset of disease and aggressive IE with congenital heart disease was discovered later. The aim of this case report is to elucidate the role of imaging modalities in detecting superimposed cardiac lesions.

## CASE REPORT

**Fig.1:**
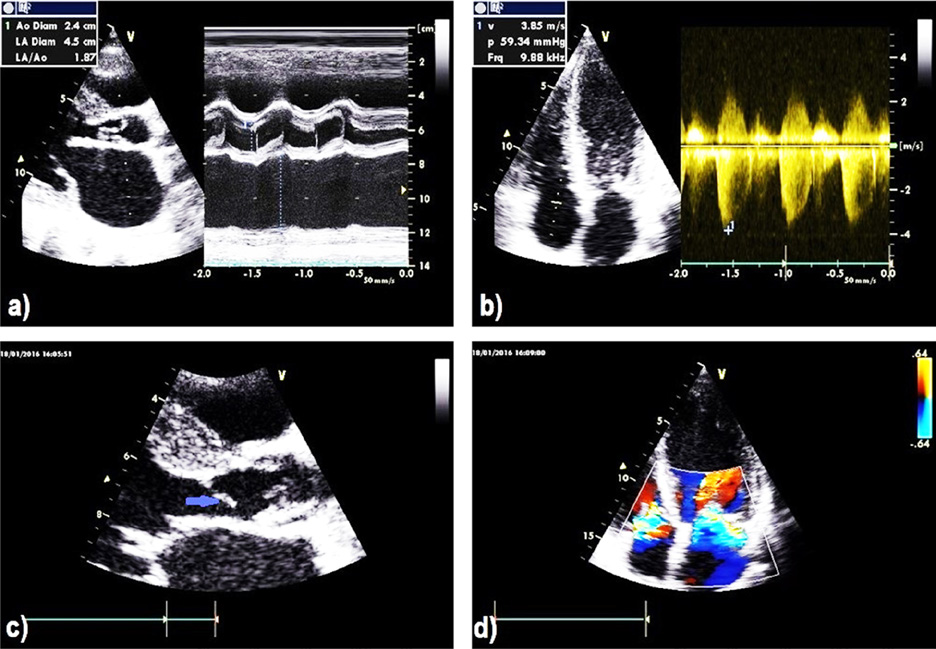
Transthoracic echocardiography (TTE) images show **a)** M-mode revealing dilated left atrium, **b)** Tricuspid regurgitation with severe pulmonary hypertension, **c)** multiple masses on the aortic valve, the longest of 20 mm implanted like a membrane (arrow), **d)** Color flow mapping (CFM) revealing severe tricuspid regurgitation (TR) and mitral regurgitation (MR).

The case involves an 18-year-old male with known rheumatic heart disease (RHD) since 11 years of age who was on irregular treatment. The patient presented with shortness of breath (SOB) for two weeks. The patient was diagnosed, admitted, and treated as decompensated heart failure and improved. After one month, the patient returned with fever and SOB. On examination, he was conscious, oriented, and cooperative, but he was ill, pale, and toxic with third degree finger clubbing. Auscultation revealed decreased air entry in the chest bilateral basally, palpable hyperdynamic apex beat of the heart shifted to the sixth intercostal with palpable systolic thrill with diastolic murmur. Chest radiograph (CXR) revealed an enlarged left atrium of the heart. Transthoracic and transesophageal echocardiography (TTE and TEE) showed a dilated left atrium (Fig.1a) and destroyed and perforated leaflets of the tricuspid valve with severe (grade IV/IV) aortic regurgitation (AR) and stenosis (Fig.1b). There were also multiple masses on the aortic valve, the longest at 20 mm implanted like a membrane on the left ventricular outflow tract with vegetation or a subarctic membrane about 14 mm away from the aortic valve (Fig.1c). There was also thickening of the mitral valve associated with severe mitral regurgitation (MR) on color flow mapping (CFM) (Fig.1d) with fenestrated anterior leaflets of the mitral valve.

### Transesophageal echocardiography

**Fig.2:**
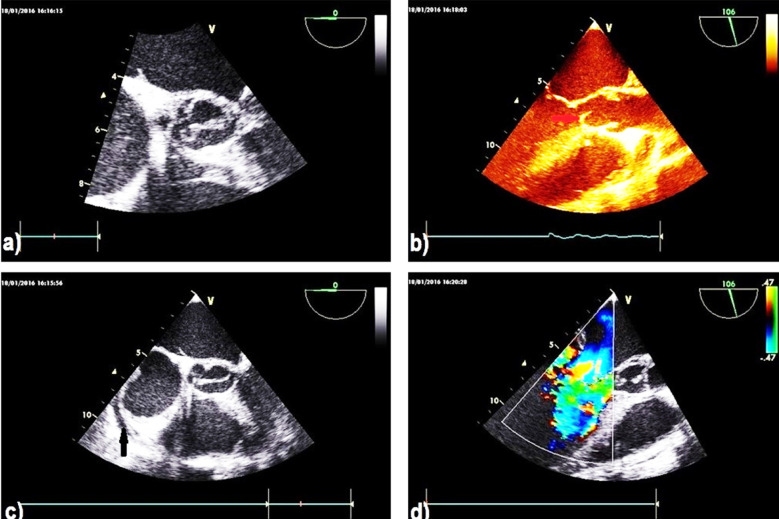
Trans esophageal echocardiography (TEE) show **a)** detracted aortic valve, **b)** the presence of sub aortic membrane (arrow), **c)** minimal pericardial effusion (arrow), **d)** severe mitral regurgitation (MR) with perforation of the anterior mitral valve leaflet.

(TEE) confirmed the above, showing detracted aortic valve leaflets (Fig.2a) and detecting the presence of a subaortic membrane (Fig.2b) and minimal pericardial effusion (Fig.2c). The presence of severe mitral regurgitation (MR) was confirmed with perforation of the anterior mitral valve leaflet (Fig.2d). There was also significant vegetation between the chordae tendinae.

The left ventricle was normal in dimension with good contractility and 63% ejection fraction (EF) apart from mild concentric hypertrophy. The right heart appeared normal in dimension apart from severe pulmonary hypertension causing moderate secondary TR (III/IV). Abdominal ultrasound imaging (US) revealed a congested and enlarged liver, inferior vena cava (IVC), and spleen. Blood culture revealed staphylococcus aureus. This was a case of aggressive IE severely destructing the aortic and mitral valves with vegetation-like masses and an incidentally discovered sub aortic valve membrane.

### Therapeutic intervention

The patient was admitted to the cardiology department and treated with excessive antibiotics according to the guidelines of European society of cardiology for one month.^7^ The patient referred for open heart surgery. Surgical resection of the sub aortic membrane was done and both aortic and mitral valves were replaced with prosthetic valves. The patient was discharged after surgery in good health.

### Follow up and outcomes

**Fig.3:**
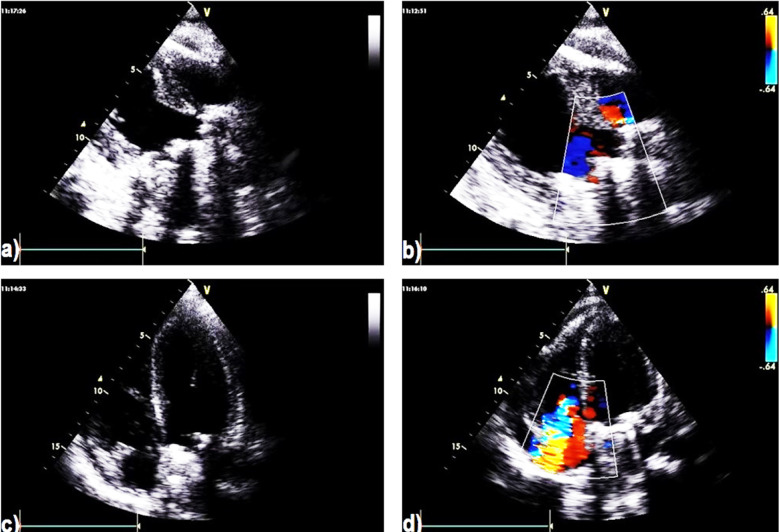
Transthoracic echocardiography (TTE) post-operative images show **a)** aortic valve replacement, **b)** Color flow mapping (CFM) showing good functioning metallic aortic and mitral valves, **c)** apical view showing good positioning of the mitral valve, **d)** Color flow mapping (CFM) revealing remnant mild tricuspid regurgitation (TR).

Post-operative TTE images show the prosthetic aortic and mitral valves (Fig.3a) with good functioning on CFM (Fig.3b) and good positioning of both (Fig.3c) however, CFM revealed the remnants of mild TR (Fig.3d). In this evaluation, the patient was a symptomatic and regular on daily warfarin.

## DISCUSSION

IE is uncommon in developed countries but more prevalent in developing countries, including countries in the Middle East. It usually presents as an ambiguous disease with a wide variety of clinical manifestations, making diagnosis difficult or resulting in a misleading or false diagnosis.^8^ The current case was primarily diagnosed as rheumatic heart disease; however, the sub aortic membrane had not been discovered primarily. The membrane was complicated by IE with multiple vegetation like masses over the aortic valve. This case is similar to the case reported by Gurel et al., who reported the presence of a well-defined relationship between sub aortic membrane and IE.^9^ Our case is also compatible with the case reported by Sari et al., who reported a case of sub aortic membrane complicated by IE and severe AR.^10^

Our case confirmed that TEE is useful as an imaging modality to diagnose papillary muscle abnormalities that may be difficult to discover by TTE even by experienced cardiologists as reported by Cahill et al., and Costa et al.^2^,^11^ In the current case, antibiotics played an important role in improving IE before surgical intervention. Ultimately, we report this case to highlight the role of medical imaging modalities in diagnosing superimposed medical lesions as in the current case where multiple superimposed aortic lesions were detected. Russell et al.,^12^ documented the importance of TEE in diagnosing subtle sub aortic membrane.

## CONCLUSION

Infective endocarditis is a devastating infection of the heart. Medical imaging plays an essential role in detecting cardiac lesions. Trans esophageal echocardiography is a highly valuable imaging modality for diagnosis of superimposed aortic valve and heart lesions in general and it is necessary to avoid a likely fatal events.

### Author’s Contribution:

**SAA** Prepared the initial and final draft of article and critically reviewed and approved the final draft and is responsible for the accuracy of the work.

**NNA** collected and organized data.

**MBG** analyzed and interpreted data.

**AMO** revised the manuscript.
